# A Prospective Study of Serum Trace Elements in Healthy Korean Pregnant Women

**DOI:** 10.3390/nu8110749

**Published:** 2016-11-23

**Authors:** Rihwa Choi, Jiyu Sun, Heejin Yoo, Seonwoo Kim, Yoon Young Cho, Hye Jeong Kim, Sun Wook Kim, Jae Hoon Chung, Soo-young Oh, Soo-Youn Lee

**Affiliations:** 1 Department of Laboratory Medicine and Genetics, Samsung Medical Center, Sungkyunkwan University School of Medicine, 81 Irwon-Ro, Gangnam-Gu, Seoul 06351, Korea; rihwa.choi@samsung.com; 2 Statistics and Data Center, Research Institute for Future Medicine, Samsung Medical Center, 81 Irwon-Ro, Gangnam-Gu, Seoul 06351, Korea; jiyu.sun@sbri.co.kr (J.S.); heejin.yoo@sbri.co.kr (H.Y.); 3 Statistics and Dater Center, Samsung Biomedical Research Institute, 81 Irwon-Ro, Gangnam-Gu, Seoul 06351, Korea; seonwoo1.kim@samsung.com; 4 Division of Endocrinology and Metabolism, Department of Medicine, Thyroid Center, Samsung Medical Center, Sungkyunkwan University School of Medicine, 81 Irwon-Ro, Gangnam-Gu, Seoul 06351, Korea; yoonung2@hanmail.net (Y.Y.C.); eyedr53@schmc.ac.kr (H.J.K.); sunwooksmc.kim@samsung.com (S.W.K.); jaeh.chung@samsung.com (J.H.C.); 5 Department of Obstetrics and Gynecology, Samsung Medical Center, Sungkyunkwan University School of Medicine, 81 Irwon-Ro, Gangnam-Gu, Seoul 06351, Korea; 6 Department of Clinical Pharmacology & Therapeutics, Samsung Medical Center, 81 Irwon-Ro, Gangnam-Gu, Seoul 06351, Korea

**Keywords:** trace elements, zinc, copper, cobalt, selenium, pregnancy

## Abstract

This prospective study sought to investigate serum levels of trace elements (cobalt, copper, zinc, and selenium) and to assess their effects on pregnancy and neonatal outcomes. Serum levels of trace elements in 245 Korean pregnant women (median gestational age at delivery was 39 + 4 weeks and interquartile range was 38 + 4–40 + 1 weeks) were compared with those of 527 general adults and those of previous studies in other ethnic groups. Pregnancy and neonatal outcomes including gestational diabetes, preeclampsia, neonatal birth weight, and congenital abnormalities were assessed. The median serum trace element concentrations of all pregnant women were: cobalt: 0.39 μg/L (interquartile range, IQR 0.29–0.53), copper: 165.0 μg/dL (IQR 144.0–187.0), zinc: 57.0 μg/dL (IQR 50.0–64.0), and selenium: 94.0 μg/L (IQR 87.0–101.0). Serum cobalt and copper concentrations were higher in pregnant women than in the general population, whereas zinc and selenium levels were lower (*p* < 0.01). Concentrations of all four trace elements varied significantly during the three trimesters (*p* < 0.05), and seasonal variation was found in copper, zinc, and selenium, but was not observed for cobalt. The prevalence of preeclampsia was significantly lower with high copper (*p* = 0.03). Trace element levels varied by pregnancy trimester and season, and alteration in copper status during pregnancy might influence pregnancy outcomes such as preeclampsia.

## 1. Introduction

Trace elements are inorganic constituents present at very low concentrations in bodily fluids (μg/dL) and tissues (mg/kg). Those found at ng/dL or μg/kg concentrations are known as *ultra trace elements* [1]. The biological effects of deficiency disease define the essential trace elements; an element is considered essential when the signs and symptoms induced by a deficient diet are uniquely reversed by an adequate supply of the particular trace element under investigation [1]. The functions of trace elements include being a structural component of a vitamin (cobalt (Co)) and being a co-factor in metalloenzymes, such as glutathione peroxidase (selenium (Se)). Zinc (Zn) and copper (Cu), catalytic components of numerous enzymes, are also structural components of other important proteins [1]. 

Among the various techniques used for trace element analyses in human biological fluids and tissues, such as flame atomic absorption spectrometry (AAS), graphite furnace AAS, inductively coupled plasma (ICP) atomic emission spectrometry, and inductively coupled plasma-mass spectrometry (ICP-MS), ICP-MS using serum and plasma specimens is widely used to determine concentrations in both normal and disease conditions, detect and designate potential toxic metals, and diagnose trace element deficiency states and trace element-related diseases, with an advantage of being able to screen multiple elements with high sensitivity [2,3]. 

Nutrient status, including the bioavailability and concentration of trace elements, is known to be influenced by many physiological and diet factors, such as soil, geographical location, food preparation, pollution, body composition, and ethnicity [4,5,6]. During pregnancy, both physiological variables and diet influence the availability of trace elements for digestion, absorption, and utilization [7]. Physiological adaptations during pregnancy create a unique demand for essential trace elements [7]. Cu absorption rises during pregnancy due to the increased need for maternal Cu-containing enzymes such as cytochrome c oxidase, which is required for aerobic respiration and superoxide dismutases, the enzymes that catalyze the dismutation of superoxides into oxygen and hydrogen peroxide [8]. Increased Cu during pregnancy interferes with the absorption of Zn and explains the low concentration of Zn [5]. Zn and Se requirements are elevated in pregnancy to provide sustenance for the fetus [9]. The demands for trace elements in a normal pregnancy, especially during the third trimester and at parturition, impose considerable systemic oxidative, metabolic, and inflammatory stresses that play an important role in many diseases and adverse pregnancy outcomes such as miscarriages, preeclampsia, gestational diabetes mellitus (GDM), and intra-uterine growth restriction [10,11,12,13,14,15,16]. There is insufficient evidence regarding the potential adverse effects of excess Co on pregnancy outcomes, although mouse models have suggested that metal-induced chromosomal changes and DNA damage could have carcinogenic effects in females and mutagenic and teratogenic effects in their offspring [17].

Although some researchers have worked to identify the association between maternal trace element status and pregnancy and infant outcomes in different populations, the function of trace elements in the etiology of pregnancy and infant outcomes has yet to be elucidated [11,18,19,20]. In addition, no reliable data have been collected for a large population-based estimation of the trace element status of Korean pregnant women. Furthermore, little information is available regarding gestational changes in trace element status in pregnant women among different trimesters or how these changes are affected by seasons despite possible associations with pregnancy and neonatal outcomes as an effect modifier of the associations [21,22,23,24]. Seasonal variation could also fluctuate in tropical and non-tropical climates [21]. 

Therefore, in this prospective study, we measured four trace elements (Co, Cu, Zn, and Se) in pregnant women in Korea using ICP-MS and compared them with the levels in a non-pregnant group. Second, we compared maternal serum trace elements based on pregnancy trimester and season, taking into account various maternal demographic characteristics. Lastly, we investigated the effect of trace element status on pregnancy and neonatal outcomes.

## 2. Methods

### 2.1. Study Population

The target population of this study was consecutive participants, comprised of pregnant women living in South Korea for each trimester of their pregnancy and visiting Samsung Medical Center from April 2012 to September 2013. During the study period, we recruited 282 pregnant women. From among them, we excluded 37 women: 3 women for a history of smoking; 6 women for a history of concurrent serious medical disease that could affect pregnancy and neonatal outcomes; 5 women for a twin pregnancy; 21 women for lack of information about pregnancy outcomes due to follow-up loss; and 2 women for a miscarriage at 10 weeks of gestational age or fetal death in utero at 19 + 4 weeks of gestational age. Thus we ultimately enrolled a total of 245 women and their babies. The serum trace element levels in these pregnant women were compared with those from 527 healthy subjects (300 males and 227 non-pregnant females, range 25–82 years) who visited a health-promotion center at the Samsung Medical Center. Samples from control subjects including non-pregnant women of reproductive age with blood chemistry results within the reference range were collected during the same period as for pregnant women.

This study was conducted according to the guidelines laid down in the Declaration of Helsinki, and all procedures involving human subjects were approved by the Institutional Review Board of the Samsung Medical Center (SMC 2011-12-041-001). The subjects provided written consent for their participation in the study.

### 2.2. Data Collection

Information about socio-demographic characteristics, smoking, alcohol consumption during pregnancy and during 4 weeks prior to the last menstrual period, concurrent medical diseases and medications, and obstetrical and gynecological histories including parity (number of deliveries) were gathered through general questionnaires at the first visit for prenatal consultation, and we obtained them from electronic medical records. For all the women included in the study, the pre-pregnancy body mass index (BMI) was obtained from the self-reported weight and height recorded during the first prenatal consultation. The first trimester BMI was used as a proxy for pre-pregnancy BMI if the pre-pregnancy body weight was unclear. Gestational age was determined according to the last menstrual period or based on the ultrasonographic finding in the first trimester. Seasons at blood sampling were categorized as spring (March–May), summer (June–August), fall (September–November) and winter (December–February). Pregnancy and neonatal outcomes were obtained from hospital medical records. 

### 2.3. Pregnancy and Neonatal Outcomes

Low birth weight was defined as a neonate birth weight of less than 2500 g (5.5 pounds), regardless of gestational age. Small for gestational age (SGA) babies were defined as those with birth weights below the 10th percentile for their gestational age as determined by birth weight percentile nomograms (National Data from Korean Health Insurance Review and Assessment Service 2009). GDM was defined according to the Carpenter and Coustan criteria [25]. Preeclampsia was defined as the new onset of hypertension (≥140/90 mm Hg on two separate occasions ≥4 h apart) and proteinuria (≥300 mg/24 h) after 20 weeks of gestation [26]. Congenital anomaly was defined when the structural anomaly of the baby was identified prenatally or at birth.

### 2.4. Laboratory Analyses

Blood samples were collected from the antecubital vein. To minimize contamination, blood was collected in the royal blue stoppered trace element tubes (catalog #369737, BD Vacutainer™ glass sterile tube, Becton Dickinson Co., Franklin Lakes, NJ, USA) used for analysis. Approximately 250 μL of serum was separated and immediately stored at −70 °C until the moment of analysis. Serum Co, Cu, Zn, and Se levels were analyzed using an Agilent 7500ce ICP-MS (Agilent Technologies, Inc., Tokyo, Japan). National Institute of Standards and Technology-traceable 10 mg/L and 1000 mg/L elemental standards were used for preparation of multi-element calibration standards. Both intra- and inter-assay imprecision were <10% of the coefficient of variation. Accuracy was assured by the Proficiency Testing/Quality Management program of the Unites States College of American Pathologists survey (CAP). The CAP survey periodically provides quality control samples, which were shared among laboratories, and acceptable criteria with target values. High or low trace element status groups were defined when the serum concentration of a subject was outside the reference range, which was determined based on our previous study in healthy Korean adults [27].

### 2.5. Statistical Analysis

Categorical variables are presented as frequency and percentage. The chi-squared test was used to compare categorical variables. The appropriateness of the sample size in this study to achieve the primary objective was verified. With the estimated standard deviation of concentration from the data for each trace element and an effect size of 0.1 for Co and 20 for Cu, Zn, and Se, the size of this study has a power higher than 90% under a 5% significance level and provides sufficient precision for the estimation of concentrations of the trace elements, as represented by confidence interval. Because age, pre-pregnancy BMI, serum Co and Zn levels, gestational age at delivery, and baby body weight at birth were not normally distributed, we used nonparametric methods. Normality for each continuous variable was checked using the Shapiro-Wilk test and a histogram. Median and interquartile range (IQR) was used as the measure of central tendency and dispersion, respectively, due to non-normality. Differences in those continuous variables among trimesters, seasons, and age groups were analyzed using the Kruskal-Wallis test. To assess the effects of demographic variables on trace elements (high or low trace element status groups), we performed simple and multiple logistic regression analyses for univariable and multivariable analyses, respectively. In the case of a rare event, we applied a logistic regression model using Firth’s penalized maximum likelihood estimation method. Variables with a *p* value of less than 0.2 in the univariable analysis were included in the multivariable analysis. 

The primary objective in this study is to estimate the concentration of each trace element and to examine whether there is a difference in the concentration of each trace element among trimesters and seasons. To analyze the association between the high or low trace element status groups and pregnancy and neonatal outcomes, we applied multiple logistic regressions for dichotomous outcomes, and multiple linear regressions for continuous outcomes, adjusting for demographic variables selected from the multivariable analysis of pregnancy and neonatal outcomes and demographic characteristics. Statistical analysis was executed using SAS version 9.4 (SAS Institute, Cary, NC, USA). *p* values were corrected by the Bonferroni method in the case of multiple testing and were considered to be significant at the level of 0.05.

## 3. Results

### 3.1. General Characteristics of the Study Population

In total, 245 Korean pregnant women participated in this study. Their median age was 32.0 years (range 24.0–43.9 years) and median pre-pregnancy BMI was 20.2 kg/m^2^ (range 16.0–29.6 kg/m^2^). Their median gestational age at delivery was 39 + 4 weeks, and the interquartile range was 38 + 4–40 + 1 weeks. Among them, 95.5% (*n* = 234) had more than 12 years of education, and more than two-thirds had indoor jobs. The baseline characteristics of the study population are summarized in Table 1.

### 3.2. Serum Trace Element Levels in Korean Pregnant Women

The median (interquartile range) serum trace element concentrations during pregnancy (*n* = 245) are summarized in Table 2. Serum concentrations of Co and Cu were significantly higher in pregnant women than in the general population, including both non-pregnant women and men, whereas Zn and Se levels were significantly lower (Table 2).

**Figure 1:**
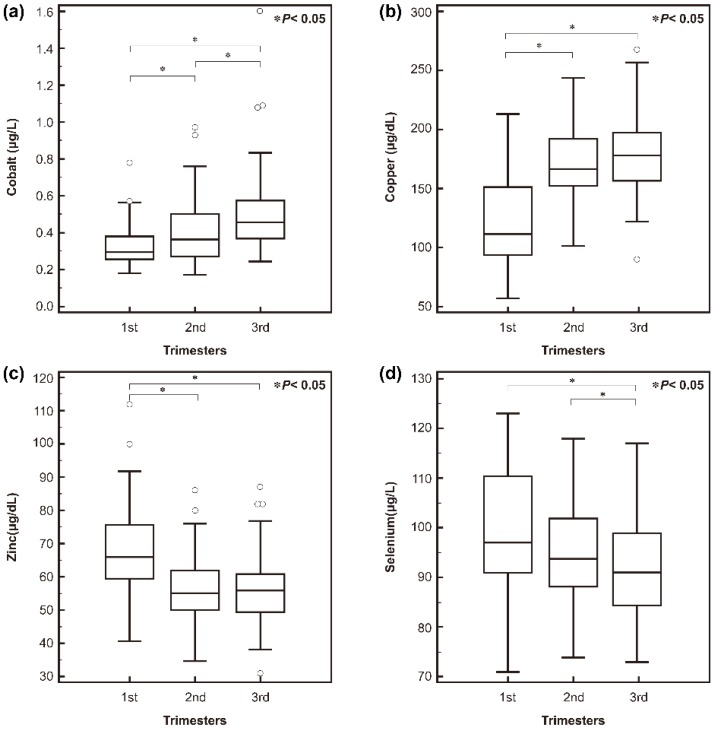
Trace element concentrations in the three trimesters: (**a**) serum cobalt; (**b**) copper; (**c**) zinc; and (**d**) selenium. All four trace element levels differed significantly by trimester.

Serum trace element concentrations, along with the demographics of the pregnant women, are shown in Table S1. In multivariable analyses, the concentrations of the four different trace elements were significantly affected by demographic factors. All four trace element levels differed significantly by trimester (Figure 1).

**Figure 2:**
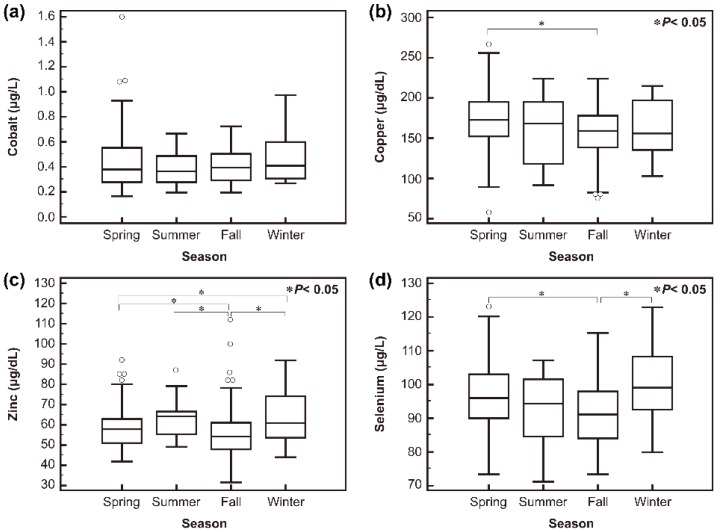
Trace element concentrations over the different seasons: (**a**) serum cobalt; (**b**) copper; (**c**) zinc; and (**d**) selenium. Although the cobalt concentration did not differ significantly by season, concentrations of copper, zinc, and selenium did.

Although the Co concentration did not differ significantly by season, concentrations of Cu, Zn, and Se did, as depicted in (Figure 2). Among the four seasons, Cu, Zn, and Se were significantly lower in fall than in spring. Zn concentrations were significantly lower in fall than in spring, summer, and winter seasons, and Se concentrations were significantly lower in fall than in spring and winter seasons. Low Cu, Zn, and Se levels were also observed in fall and winter compared to in spring and summer (Figure S1). The concentration of Co was significantly lower in women with a previous history of complicated pregnancy. The serum Zn concentration was the most variable among the four trace elements across age, trimester, season, duration of education, and job.

### 3.3. Prevalence of High or Low Trace Elements Status in Korean Pregnant Women

The overall prevalence of high or low trace element status (levels outside the reference ranges, 0.13–0.73 μg/L for Co, 55–150 μg/dL for Cu, 65–125 μg/dL for Zn, and 75–200 μg/L for Se, respectively [27]) of all participants (*n* = 245) was as follows: high Co, 3.7% (*n* = 9); high Cu, 70.2% (*n* = 172); low Zn, 76.3% (*n* = 187); and low Se, 2.0% (*n* = 5). No women had low Co or Cu or high Zn or Se levels. The numbers and percentages of high or low status across different demographic groups are shown in Table S2. The prevalence of high Cu and low Zn differed across seasons.

### 3.4. Association between High or Low Trace Element Status and Pregnancy and Neonatal Outcomes

Among the 245 pregnant women, 18 (7.3%) women had GDM, and 5 (2.0%) experienced and were managed for preeclampsia. The median gestational age at delivery was 39 + 4 weeks (IQR: 38 + 4–40 + 1 weeks). The median body weight of all delivered babies (*n* = 245) was 3180 g (IQR: 2928–3440 g). The prevalence of low birth weight and SGA neonates was 3.7% (*n* = 9) and 14.7% (*n* = 36), respectively. There were 11 (4.5%) preterm babies, and 14 (5.7%) babies were born with congenital anomalies.

The association between high or low trace element status and adverse pregnancy outcomes is summarized in Table 3. Statistical analyses were performed as pooled analysis of all trimesters. However, there was no significant association between high or low trace element status and pregnancy and neonatal outcomes when trimesters were included in the statistical analyses. In a pooled analysis, the prevalence of preeclampsia was significantly lower with high Cu (*p* = 0.03). No significant associations were observed between high Co, low Zn, or low Se and other pregnancy outcomes (GDM, gestational age at delivery, SGA, preterm birth, and congenital anomaly) using either simple or multiple logistic regression analyses.

## 4. Discussion

In this study, we investigated trace element concentrations in pregnant women as compared with the general population. Although previous studies have reported reference ranges for trace element concentrations in healthy pregnant women using blood samples [8,23,28,33], only a few studies have reported those concentrations in comparison with those in a general population of healthy non-pregnant women or healthy men [8,23,33]. Comparisons of trace element concentrations in healthy pregnant women in different previous studies are summarized in Table 4. Researchers in different countries using different detection methods have endeavored to establish reference levels for trace elements in healthy pregnant women while considering the physiological changes that occur during pregnancy. Those previous studies were performed with variable specimen types (serum, plasma, or whole blood) and determined the concentrations using an AAS method other than ICP-MS, which made direct comparison of trace element concentrations difficult. In the present study, the serum concentrations of Co and Cu were higher in pregnant women than in the general population, whereas Zn and Se levels were lower. Those results are similar to those of previous studies in Turkish, Chinese, and Chilean populations [8,23,33], except for Co because no previous study compared Co concentrations in pregnant women with those in the general population.

Several previous studies in different geographic regions examining trace element concentrations in pregnant women used ICP-MS, but they did not compare the results with those of the general population. The median concentration of serum Co in pregnant women evaluated in this study was higher than in Canadian (mean 0.24 μg/L in plasma) and western Australian pregnant women (mean 0.23 μg/L in whole blood) [34,35] and lower than in Israeli (mean 0.74 μg/L in plasma) and Iranian pregnant women (mean 0.54 μg/L in whole blood) [36,37]. Compared with the results of other studies, the median value of Zn in Korean pregnant women was lower than that of pregnant women in other populations, whereas the levels for other trace elements were comparable. Because the adequacy of micronutrient intake and consequent blood concentration during pregnancy have also been suggested to be affected by environmental, cultural, and demographic variables, it is crucial to understand at a population level which nutrients from food intake are limited and whether efforts are required to optimize maternal nutrient intake, including supplementation or food fortification [38]. Differences in trace element concentration could result from soil, geographical location, food preparation and processing, food accessibility, cultural practices, pollution, or ethnic differences in body composition and genetics [7]. A recent genome-wide association study has identified loci affecting blood Cu, Se, and Zn from 2603 Australian and 2874 British participants [4].

In the present study, serum trace element levels differed significantly by trimester; Co and Cu increased during pregnancy, and the concentration of Zn and Se decreased. Those results are compatible with those of previous studies done in northern Norway, Iran, Turkey, China, and Spain [8,28,32,39,40]. Cu absorption rises during pregnancy due to the increased need for maternal Cu-containing enzymes such as cytochrome c oxidase, which is required for aerobic respiration, and superoxide dismutases, the enzymes that catalyze the dismutation of superoxides into oxygen and hydrogen peroxide [8]. Increased Cu during pregnancy interferes with the absorption of Zn and thus explains the low concentration of Zn [5]. Zn is widely recognized for its critical roles in cell division, differentiation, and function that are essential for tissue growth. Consequently, Zn is a key nutrient during embryogenesis, fetal growth, and development, which increase the mother’s Zn needs during pregnancy [41]. Plasma Zn concentrations stabilize at normal or near-normal concentrations under conditions of chronic low Zn intake because of changes in gastrointestinal absorption and excretion, with months (rather than weeks) required for complete equilibration of all extra-plasma Zn pools to occur [41]. A study done in Spain found that an early and progressive decline in serum Zn occurs during gestation, and poor maternal Zn status could limit the metabolic adaptation capacity of women, especially during pregnancy [32].

Low Se concentrations in the environment and dietary intake could affect the blood concentration [7]. Decreasing blood Se concentrations have been observed and reported in regions with low dietary intake of Se, such as Poland, Yugoslavia, and Peru [42,43,44]. In addition, those studies found elevated Se requirements as pregnancy progressed to provide sustenance for the fetus [42,43,44], a trend that was not observed in regions with high soil Se content and adequate dietary intake, such as southern Spain and the southeastern Mediterranean region of Turkey [8,32]. Serum concentrations of Zn and Se are significantly lower in the fall, which suggests that the data might reflect dietary changes attributable to the availability of different foodstuffs in different seasons [23]. Although we did not assess dietary intakes, this discrepancy could result from different dietary patterns between women with different education levels [45]. In this study, Cu concentrations were lower in fall and winter seasons than in spring and summer seasons and the prevalence of preeclampsia was higher in pregnant women with normal Cu status (4.1%) than in those with high Cu status (1.2%). Previous studies about altered Cu concentrations in association with preeclampsia in pregnant women have shown inconsistent results. Although lower Cu concentrations in preeclamptic women than in a control group (pregnant women with normal blood pressure) have been reported in Saudi Arabian and Bangladeshi populations, showing results similar to the current study [18,46], a recent meta-analysis showed higher Cu levels in preeclamptic women than in healthy pregnant women, in contrast to the current study [47]. Meanwhile, considering a previous systematic review about the association of seasonality and hypertension in pregnancy indicating that the prevalence of gestational hypertension is highest during the winter months in non-tropical regions [21], the results of our study could be interpreted in the same context, suggesting that seasonal variation in Cu status was associated with preeclampsia. Although the reasons for these patterns remain unclear, seasonal variation in infectious diseases, environmental triggers of asthma, vitamin D levels, physiological responses to cold temperatures, healthcare access, and nutritional intake may all play a role [21]. One possibility for the seasonal variation in trace element status is the differences in dietary intake; it has been reported that seafood intake such as oysters, which are known to be Zn- and Se-rich, is higher in winter than in summer in Korean non-pregnant adults [9,48,49]. Decreased Cu concentrations in winter could be due to reduced Cu absorption by high Zn concentration in the intestine [1]. However, because no dietary information was assessed in the current study, future studies including that information are needed. Also, differences in Cu concentrations should be considered with caution because the seasonal variation was quite small, even though statistically significant differences were observed. Future studies comprising large populations and the aforementioned factors are needed to clarify the clinical significance of alteration of Cu concentrations.

In this study, trace element statuses were not significantly different in pregnant women delivering SGA or non-SGA babies. Previous studies about clinical significance of decreased concentrations of trace elements in association with SGA babies were inconsistent. A recent systematic study about the effect of maternal nutrition before and during early pregnancy on maternal and infant outcomes concluded that the overall quality of evidence of consistency for birth weight, low birth weight (<2500 g), SGA, and preterm delivery was low, although several studies have reported decreased trace element concentrations or deficiencies in association with SGA [50,51,52].

In this study, low serum Zn (<65 μg/dL) was observed in 76.3% (187/245) of pregnant women. Maternal Zn deficiencies produce effects ranging from infertility and embryo/fetal death to intrauterine growth retardation and teratogenesis. Post-natal complications of maternal Zn deficiency can also occur, including behavioral abnormalities, impaired immunocompetence, and an elevated risk of high blood pressure in the offspring [10,53]. We found no association between low Zn in maternal serum and pregnancy outcomes. One study on maternal-neonatal Zn serum concentrations indicated that Zn levels were significantly decreased after vaginal delivery suggesting that lower Zn levels post-vaginal delivery could be due to the participation of skeletal and uterine muscle in Zn redistribution [54]. Those studies might indirectly suggest a possible role for maternal Zn in active labor, and thus sustained Zn deficiency during pregnancy could affect the pregnancy outcome in the form of labor abnormalities [55]. However, a recent systematic review of randomized controlled trials reported no clear differences between the Zn supplementation and no Zn supplementation groups for any primary maternal or neonatal outcomes except the induction of labor in a single trial [56]. The evidence for a 14% relative reduction in preterm birth with Zn supplementation compared with placebo was primarily found in trials involving women of low income, which has some relevance in areas of high perinatal mortality, but no convincing evidence supported the idea that Zn supplementation during pregnancy results in other useful or important benefits [56]. The United Nations Children’s Fund already promotes antenatal use of multiple-micronutrient supplementation, including Zn, for all pregnant women in developing countries. In Korea, a cross-sectional study on the association between Zn and Cu intakes mid-pregnancy (12–28 weeks of gestation) and birth weight and height found that Zn intake was positively associated with birth weight and height [57]. The median Zn intake among 918 Korean pregnant women was 8.4 mg/day, which is lower than the Institute of Medicine’s 2006 suggested dietary reference intakes for estimated average requirements (9.5 mg/day for pregnant women aged 19–50 years) and recommended dietary allowances and adequate intakes (11 mg/day for pregnant women aged 19–50 years) [9]. Another study noted significant positive relationships between maternal Zn intake from animal food sources and birth weight and height [57]. However, maternal Zn status was not assessed in that study. Future studies that collect information about both maternal Zn status and dietary intake from a large sample are needed to elucidate the clinical effects of Zn during pregnancy. 

The strengths of our study include its prospective design and the use of the gold-standard ICP-MS method to measure multiple trace elements simultaneously in pregnant women. We also assessed trace element concentrations across pregnancy trimester and season and identified a high prevalence of Zn deficiency in Korean pregnant women which could provide background information for a study on Zn supplementation.

Although we assessed several demographic characteristics, some factors important for determining trace element status, such as dietary intake, were not assessed. Another important limitation of this study is that manganese and other proteins involved in the superoxide dismutase antioxidant pathways such as selenoprotein P and glutathione peroxidase enzymes, which might have potential mechanisms relating to the altered trace element concentrations, were not measured in this study. Concentrations of ceruloplasmin, the major Cu-carrying protein, were also not measured. Future studies including comprehensive measurements of those other elements are needed.

## 5. Conclusions

In conclusion, low Zn status is highly prevalent (76.3%) in Korean pregnant women. Considering that Zn concentration decreases as pregnancy progresses, nutrient supplementation might be needed for Korean pregnant women. Our study also indicates that high Cu is associated with a lower prevalence of preeclampsia (1.2% versus 4.1%). However, future studies to assess the associations among nutrient supplementation, maternal serum concentration, and pregnancy-related outcomes are needed. This study could provide important guidance for the reasonable supplementation of essential elements in pregnancy to improve maternal and neonatal outcomes.

## Figures and Tables

**Table 1:** Demographic characteristics of the sample of 245 Korean pregnant women.

Characteristics	Total (*n* = 245)		1st Trimester (*n* = 52)		2nd Trimester (*n* = 97)		3rd Trimester (*n* = 96)		*p*
	*n*	%	*n*	%	*n*	%	*n*	%	
Season									0.10
Spring	110	44.9%	22	20.0%	39	35.5%	49	44.5%	
Summer	25	10.2%	6	24.0%	10	40.0%	9	36.0%	
Fall	94	38.4%	18	19.1%	39	41.5%	37	39.4%	
Winter	16	6.5%	6	37.5%	9	56.3%	1	6.3%	
Education level									1.0
≤12 years	11	4.5%	2	18.2%	5	45.5%	4	36.4%	
>12 years	234	95.5%	50	21.4%	92	39.3%	92	39.3%	
Jobs									0.94
Any job	167	68.2%	36	21.6%	67	40.1%	64	38.3%	
Homemaker	78	31.8%	16	20.5%	30	38.5%	32	41.0%	
Type of current pregnancy									0.15
Spontaneous pregnancy	239	97.6%	52	21.8%	92	38.5%	95	39.7%	
Artificial pregnancy	6	2.4%	0	0.0%	5	83.3%	1	16.7%	
Parity									0.53
0 (nullipara)	151	61.6%	32	21.2%	56	37.1%	63	41.7%	
≥1	94	38.4%	20	21.3%	41	43.6%	33	35.1%	

Fisher’s exact test was used to compare categorical variables. A *p* < 0.05 was considered statistically significant.

**Table 2:** Trace element concentrations of 245 Korean pregnant women in comparison with 527 healthy non-pregnant Korean subjects (300 men and 227 women).

	Pregnant Women (*n* = 245)		Healthy Non-Pregnant Women (*n* = 227)		Healthy Men (*n* = 300)		*p* ^a^
	Median	Interquartile Range	Median	Interquartile Range	Median	Interquartile Range	
Cobalt (μg/L) ^bcd^	0.39	(0.29–0.53)	0.36	(0.25–0.51)	0.30	(0.18–0.45)	<0.0001
Copper (μg/dL) ^bcd^	165.0	(144.0–187.0)	100.0	(86.3–120.0)	91.5	(81.0–105.5)	<0.0001
Zinc (μg/dL) ^bc^	57.0	(50.0–64.0)	92.0	(84.0–104.8)	92.0	(82.0–103.0)	<0.0001
Selenium (μg/L) ^bcd^	94.0	(89.0–101.0)	140.0	(120.0–171.8)	150.0	(127.5–179.0)	<0.0001

Serum concentrations of the four trace elements differed significantly among the three populations. Among a total of 245 pregnant women, 52 were in their first trimester, 97 were in their second trimester, and 96 were in their third trimester. It is of note that the cobalt and copper concentrations of healthy pregnant women were higher than those of healthy men and women, whereas zinc and selenium concentrations were lower in healthy pregnant women than in healthy men and women. ^a^ *p* value is result of the Kruskal-Wallis test; ^b^ Post hoc analysis revealed statistically significant differences between pregnant women and healthy men (*p* < 0.05); ^c^ Post hoc analysis revealed statistically significant differences between pregnant women and healthy non-pregnant women (*p* < 0.05); ^d^ Post hoc analysis revealed statistically significant differences between healthy men and healthy non-pregnant women (*p* < 0.05).

**Table 3:** Associations between high or low trace element status and pregnancy and neonatal outcomes.

Pregnancy and Neonatal Outcomes	Cobalt (μg/L)				Copper (μg/dL)				Zinc (μg/dL)				Selenium (μg/L)			
	High (*n* = 9)	Not High (*n* = 236)	*p* ^a^	*p* ^b^	High (*n* = 172)	Not High (*n* = 73)	*p* ^a^	*p* ^b^	Low (*n* = 187)	Not Low (*n* = 58)	*p* ^a^	*p* ^b^	Low (*n* = 5)	Not Low (*n* = 240)	*p* ^a^	*p* ^b^
Gestational diabetes, *n* (%)	0 (0)	18 (7.6)	0.76	0.80	15 (8.7)	3 (4.1)	0.22	0.45	15 (8.0)	3 (5.2)	0.47	0.58	0 (0)	18 (7.5)	0.96	0.79
Preeclampsia, *n* (%)	0 (0)	5 (2.1)	0.62	0.43	2 (1.2)	3 (4.1)	0.16	**0.03**	5 (2.7)	0 (0)	0.40	0.54	0 (0)	5 (2.1)	0.42	0.44
Gestational age at delivery, weeks, median	39.6	39.5	0.43	0.27	39.4	39.6	0.37	0.99	39.6	39.5	0.88	0.44	39.6	39.4	0.39	0.64
Baby weight, g, median	3540	3180	0.08	**0.03**	3180	3190	0.76	0.47	3180	3175	0.59	0.30	3440	3180	0.04	**0.03**
Preterm, *n* (%)	0 (0)	11 (4.7)	0.98	0.65	10 (5.8)	1 (1.4)	0.16	0.18	7 (3.7)	4 (6.9)	0.32	0.66	0 (0)	11 (4.6)	0.72	0.64
Low birth weight, *n* (%)	0 (0)	9 (3.8)	0.88	0.94	6 (3.5)	3 (4.1)	0.81	0.72	8 (4.3)	1 (1.7)	0.38	0.56	0 (0)	9 (3.8)	0.63	0.97
Small for gestational age, *n* (%)	1 (11.1)	35 (14.8)	0.76	0.40	27 (15.7)	9 (12.3)	0.50	0.80	31 (16.6)	5 (8.6)	0.14	0.19	0 (0)	36 (15.0)	0.68	0.50
Congenital abnormality, *n* (%)	0 (0)	14 (5.9)	0.89	0.83	10 (5.8)	4 (5.5)	0.92	0.84	11 (5.9)	3 (5.2)	0.84	0.94	0 (0)	14 (5.8)	0.83	0.95

^a^ *p* values for univariable analysis; ^b^ *p* values for multivariable analysis; High or low trace element status groups were defined as a serum concentration outside the reference range. References ranges are: for serum cobalt concentration 0.13–0.73 μg/L, for serum copper concentration 55–150 μg/dL, for serum zinc concentration 65–125 μg/dL, and for serum selenium concentration 75–200 μg/L. No pregnant women with low cobalt or low copper or high zinc or high selenium were observed in our study population. To analyze the association between high or low trace element status groups and adverse pregnancy outcomes, we applied multiple logistic regressions for dichotomous outcomes and multiple linear regressions for continuous outcomes, adjusting for demographic variables selected from the univariable analysis of adverse pregnancy outcomes and demographic characteristics. In the case of a rare event, we applied a logistic regression model using Firth’s penalized maximum likelihood estimation method. Bold = significant at *p* < 0.05 for multivariable analysis.

**Table 4:** Trace element concentrations in healthy pregnant women compared with those in healthy non-pregnant women and healthy men.

	Ethnicity	Method	Specimen	Preg All	1st Trimester		2nd Trimester		3rd Trimester		Healthy Non-Pregnant Women		Healthy Men		Reference
				*n*	*n*	conc.	*n*	conc.	*n*	conc.	*n*	conc.	*n*	conc.	
Cobalt (μg/L)	Korean	ICP-MS	Serum	245	52	0.30 ^a^	97	0.36 ^a^	96	0.46 ^a^	227	0.36 ^a^	300	0.30 ^a^	This study
Copper (μg/dL)	Korean	ICP-MS	Serum	245	52	112.0 ^a^	97	167.0 ^a^	96	178.0 ^a^	227	100.0 ^a^	300	91.5 ^a^	This study
	Iranian	ICP-MS	Serum	162	162	130.9 ± 43.5	162	172 ± 38.94	162	193.2 ± 28.5	-	-	-	-	[28]
	USA ^d^	ICP-OES	Serum	44	44	168.5 ± 5.18 ^b^	-	-	-	-	-	-	-	-	[29]
	Jordanian	AAS	Serum	186	52	175 ± 42	42	226 ± 51	92	236 ± 36	-	-	-	-	[30]
	Turkish	AAS	Serum	351	177	132.33 ± 38.24	174	164.86 ± 39.69	-	-	30	104.75 ± 39.14	30	78.29 ± 20.90	[8]
	Spanish	AAS	Serum	52	52	217.1 ± 4.9 ^b^	-	-	-	-	50	106.9 ± 2.8 ^b^	-	-	[31]
	Spanish	AAS	Serum	159	73	147.5 ± 34.6	30	197.1 ± 24.0	GA 25–35 wk: 18 GA >35 wk: 38	195.1 ± 35.0 204.2 ± 41.8	-	-	-	-	[32]
	Chinese	AAS	Blood	2380	550	102.6 ^a^	GA 13–20 wk: 552 GA 21–27 wk: 600	130.2 ^a^ 135.6 ^a^	GA 28–35 wk: 553 GA 36–42 wk: 125	136.6 ^a^ 145.2 ^a^	552	83.5 ^a^	-	-	[33] ^c^
	Chilean	AAS	Plasma	98	-	-	-	-	GA 27–32 wk: 34 GA 33–35 wk: 47 GA >36 wk: 45	203.0 ± 39.9 217.7 ± 37.2 218.2 ± 45.2	29	131.0 ± 21.2	-	-	[23] ^c^
Zinc (μg/dL)	Korean	ICP-MS	Serum	245	52	66.0 ^a^	97	55.0 ^a^	96	94.0 ^a^	227	92.0 ^a^	300	92.0 ^a^	This study
	Iranian	ICP-MS	Serum	162	162	79.5 ± 15	162	74.5 ± 16.1	162	65.3 ± 14.9	-	-	-	-	[28]
	USA ^d^	ICP-OES	Serum	44	44	78.9 ± 2.15 ^b^	-	-	-	-	-	-	-	-	[29]
	Jordanian	AAS	Serum	186	52	77.0 ± 16.0	42	73.0 ± 14.0	92	68.0 ± 10.0	-	-	-	-	[30]
	Turkish	AAS	Serum	351	177	81.30 ± 31.94	174	74.25 ± 22.47	-	-	30	121.41 ± 29.22	30	134.85 ± 15.95	[8]
	Spanish	AAS	Serum	159	73	71.3 ± 12.9	30	61.1 ± 8.6	GA 25–35 wk: 18 GA >35 wk: 38	59.8 ± 10.0 58.5 ± 11.5	-	-	-	-	[32]
	Chinese	AAS	Blood	2380	550	83.3 ^a^	GA 13–20 wk: 552 GA 21–27 wk: 600	81.75 ^a^ 78.95 ^a^	GA 28–35 wk: 553 GA 36–42 wk: 125	79.95 ^a^ 81.2 ^a^	552	83.1 ^a^	-	83.1 ^a^	[33]
	Chilean	AAS	Plasma	98	-	-	-	-	GA 27–32 wk: 34 GA 33–35 wk: 47 GA >36 wk: 45	105.9 ± 33.3 110.1 ± 34.1 117.0 ± 23.6	29	128.8 ± 21.0	-	-	[23] ^c^
Selenium (μg/L)	Korean	ICP-MS	Serum	245	52	97.0 ^a^	97	94.0 ^a^	96	91.0 ^a^	227	140.0 ^a^	300	150.0 ^a^	This study
	Turkish	AAS	Serum	351	177	44.85 ± 9.23	174	47.18 ± 10.92	-	-	30	55.38 ± 8.81	30	72.24 ± 9.28	[8]
	Spanish	AAS	Serum	159	73	108.6 ± 20.1	30	99.0 ± 24.4	GA 25–35 wk: 18 GA >35 wk: 38	87.1 ± 16.1 85.5 ± 12.8	-	-	-	-	[32]
	UK Whites	AAS	Serum	27	-	-	-	-	Before delivery: 27	58.4 ± 14.9	22	69.8 ± 11.7	-	-	[15]
	Chilean	AAS	Plasma	98	-	-	-	-	GA 27–32 wk: 34 GA 33–35 wk: 47 GA >36 wk: 45	100.0 ± 16.5 90.6 ± 15.7 85.1 ± 19.7	29	112.6 ± 26.8	-	-	[23] ^c^

Abbreviations: AAS, atomic absorption spectroscopy; conc., concentration; GA, gestational age; ICP-MS, inductively coupled plasma mass spectrometry; ICP-OES, inductively coupled plasma optical emission spectrometry; preg, pregnant women; wk, weeks. ^a^ Median value (data were not normally distributed); ^b^ means ± standard error of means; concentrations without superscript were reported as means ± standard deviations; ^c^ Reported value in the literature was converted to μg/dL or μg/L for comparison; to convert the copper values to μg/dL, the values of μmol/L were divided by 0.1574; to convert the zinc values μg/dL to, the values of μmol/L were divided by 0.1530; to convert the selenium values to μg/L, the values of μmol/L were divided by 0.0127; ^d^ 81.8% of women were Caucasian.

## Supplementary Materials

The following are available online at http://www.mdpi.com/2072-6643/8/11/749/s1, Figure S1: Trace element concentrations over the different seasons. Low copper, zinc, and selenium levels were also observed in fall and winter compared to in spring and summer, Table S1: Association of demographic characteristics and trace element concentrations in 245 Korean pregnant women, Table S2: Association of demographic factors and high or low status of the trace elements in 245 Korean pregnant women.

## Abbreviations

The following abbreviations are used in this manuscript:

AAS, atomic absorption spectroscopy; BMI, body mass index; Co, cobalt; Cu, copper; GDM, gestational diabetes mellitus; ICP, inductively coupled plasma; ICP-MS, inductively coupled plasma-mass spectrometry; IQR, interquartile range; LMP, last menstrual period; Se, selenium; SGA, small for gestational age; Zn, zinc.
