# Supplementary material for: A Prospective Study of Serum Trace Elements in Healthy Korean Pregnant Women
Source: Nutrients. 2016 Nov 23;8(11):749. doi: 10.3390/nu8110749 (PMC5133131; doi:10.3390/nu8110749)
Supplement: Supplementary file 1 [file nutrients-08-00749-s001.docx]

Supplementary Materials: A Prospective Study of Serum Trace Elements in Pregnant Women in Relation to Pregnancy and Neonatal Outcomes

Rihwa Choi, Jiyu Sun, Heejin Yoo, Seonwoo Kim, Yoon Young Cho, Hye Jeong Kim, Sun Wook Kim, Jae Hoon Chung, Soo-young Oh and Soo-Youn Lee

**Table S1.** Association of demographic characteristics and trace element concentrations in 245 Korean pregnant women.

|  | **Cobalt Levels (µg/L)** | | | **Copper Levels (µg/dL)** | | | **Zinc Levels (µg/dL)** | | | **Selenium Levels (µg/L)** | | |
| --- | --- | --- | --- | --- | --- | --- | --- | --- | --- | --- | --- | --- |
|  | **Median (IQR)** | ***p* ^a^** | ***p* ^b^** | **Median (IQR)** | ***p* ^a^** | ***p* ^b^** | **Median (IQR)** | ***p* ^a^** | ***p* ^b^** | **Median (IQR)** | ***p* ^a^** | ***p* ^b^** |
| Trimester ^e^ |  | **0.00 ^d^** | **0.00 ^d^** |  | **0.00 ^d^** | **0.00 ^d^** |  | **0.00 ^d^** | **0.00 ^d^** |  | **0.00 ^d^** | **0.00 ^d^** |
| 1st | 0.30 (0.26–0.38) |  |  | 112.0 (94.0–151.5) |  |  | 66.0 (59.5–75.5) |  |  | 97.0 (91.0–110.5) |  |  |
| 2nd | 0.36 (0.27–0.50) |  |  | 167.0 (152.0–191.0) |  |  | 55.0 (50.0–61.0) |  |  | 94.0 (89.0–102.0) |  |  |
| 3rd | 0.46 (0.37–0.58) |  |  | 178.0 (157.0–197.5) |  |  | 56.0 (49.5–61.0) |  |  | 91.0 (84.5–99.0) |  |  |
| Season |  | 0.59 | - |  | **0.03 ^f^** | **0.00 ^cf^** |  | **0.02 ^g^** | **0.00 ^cg^** |  | **0.00 ^cfh^** | **0.00 ^cfh^** |
| Spring | 0.39 (0.28–0.55) |  |  | 173.5 (152.0–196.0) |  |  | 58.0 (51.0–63.0) |  |  | 96.0 (90.0–103.0) |  |  |
| Summer | 0.39 (0.27–0.48) |  |  | 169.0 (112.0–194.0) |  |  | 64.0 (56.0–66.0) |  |  | 94.0 (85.0–101.0) |  |  |
| Fall | 0.40 (0.29–0.50) |  |  | 159.5 (139.0–177.0) |  |  | 54.5 (48.0–61.0) |  |  | 91.0 (84.0–98.0) |  |  |
| Winter | 0.42 (0.30–0.60) |  |  | 156.5 (135.5–198.0) |  |  | 60.5 (53.5–74.0) |  |  | 99.0 (92.5–108.0) |  |  |
| Education level | | **0.03** | **0.02** |  | **0.09** | 0.10 |  | **0.04** | **0.04** |  | 0.92 | - |
| ≤12 years | 0.49 (0.39–0.68) |  |  | 187.0 (155.0–210.0) |  |  | 64.0 (61.0–74.0) |  |  | 95.0 (87.0–100.5) |  |  |
| >12 years | 0.38 (0.28–0.50) |  |  | 164.0 (144.0–187.0) |  |  | 57.0 (50.0–63.0) |  |  | 94.0 (87.0–102.0) |  |  |
| Jobs |  | 0.76 | - |  | 0.31 | - |  | **0.07** | **0.00 ^a^** |  | 0.55 | - |
| Any job | 0.41 (0.28–0.55) |  |  | 164.0 (139.0–187.0) |  |  | 57.0 (50.0–64.0) |  |  | 94.0 (88.0–101.0) |  |  |
| Homemaker | 0.39 (0.30–0.48) |  |  | 167.5 (151.0–188.0) |  |  | 58.0 (52.0–64.0) |  |  | 94.0 (86.0–102.0) |  |  |
| Type of current pregnancy | | 0.79 | - |  | **0.14** | 0.30 |  | 0.95 | - |  | **0.09** | **0.04** |
| Spontaneous | 0.39 (0.29–0.53) |  |  | 164.0 (144.0–187.0) |  |  | 57.0 (51.0–65.0) |  |  | 94.0 (87.0–101.0) |  |  |
| Artificial | 0.32 (0.30–0.60) |  |  | 189.0 (151.0–208.0) |  |  | 57.5 (48.0–58.0) |  |  | 104.5 (90.0–111.0) |  |  |
| Parity |  | 0.26 | - |  | **0.01** | **0.00 ^c^** |  | **0.07** | 0.06 |  | 0.76 | - |
| 0 (nullipara) | 0.37 (0.28–0.50) |  |  | 163.0 (137.0–184.0) |  |  | 58.0 (51.0–65.0) |  |  | 95.0 (88.0–101.0) |  |  |
| ≥1 | 0.42 (0.30–0.54) |  |  | 171.5 (154.0–194.0) |  |  | 57.0 (49.0–62.0) |  |  | 92.0 (86.0–102.0) |  |  |

Abbreviations: IQR, interquartile range; Uni-*p*, non-adjusted *p* values for univariable analysis; Multi-*p*, adjusted *p* values for multivariable analysis. To assess the effects of demographic variables on trace element concentrations (continuous variables), we performed simple and multiple linear regression analyses for univariable and multivariable analyses, respectively. Logarithmic transformation was used for cobalt and zinc with skewed distributions. Variables with a *p* value of less than 0.2 in the univariable analysis were included in the multivariable analysis. Univariable analysis revealed that age was significantly associated with cobalt, copper, and zinc concentrations (*p* < 0.01) and pre-pregnancy body mass index was associated with copper concentrations (*p* = 0.02). Multivariable analysis revealed that age was significantly associated with cobalt and zinc concentrations (*p* < 0.05) and pre-pregnancy body mass index was not associated with concentrations of any of four trace elements (*p* > 0.05). ^a^ *p* values for univariable analysis; **^b^** *p* values for multivariable analysis; ^c^ *p* < 0.01; ^d^ *p* < 0.0001; ^e^ Trace element concentrations were significantly different between the 1st and 2nd trimesters and between the 1st and 3rd trimesters; ^f^ Significantly different between spring and fall; ^g^ Significantly different between summer and fall; ^h^ Significantly different between winter and fall.

**Table S2.** Association of demographic factors and high or low status of the trace elements in 245 Korean pregnant women.

|  | **Cobalt (µg/L)** | | | | **Copper (µg/dL)** | | | | **Zinc (µg/dL)** | | | | **Selenium (µg/L)** | | | |
| --- | --- | --- | --- | --- | --- | --- | --- | --- | --- | --- | --- | --- | --- | --- | --- | --- |
|  | **>0.73  (*n* = 9)** | **≤0.73  (*n* = 236)** | ***p* ^a^** | ***p* ^b^** | **>150  (*n* = 172)** | **≤150  (*n* = 73)** | ***p* ^a^** | ***p* ^b^** | **<65  (*n* = 187)** | **≥65  (*n* = 58)** | ***p* ^a^** | ***p* ^b^** | **<75  (*n* = 5)** | **≥75  (*n* = 240)** | ***p* ^a^** | ***p* ^b^** |
| Trimester |  |  | 0.57 | - |  |  | **0.00** ^c^ | **0.00**^c^ |  |  | **0.00** ^c^ | **0.00** ^c^ |  |  | 0.61 | - |
| 1st | 1 (1.9%) | 51 (98.1%) |  |  | 14 (26.9%) | 38 (73.1%) |  |  | 24 (46.2%) | 28 (53.8%) |  |  | 1 (1.9%) | 51 (98.1%) |  |  |
| 2nd | 3 (3.1%) | 94 (96.9%) |  |  | 77 (79.4%) | 20 (20.6%) |  |  | 81 (83.5%) | 16 (16.5%) |  |  | 1 (1.0%) | 96 (99.0%) |  |  |
| 3rd | 5 (5.2%) | 91 (94.8%) |  |  | 81 (84.4%) | 15 (15.6%) |  |  | 82 (85.4%) | 14 (14.6%) |  |  | 3 (3.1%) | 93 (96.9%_ |  |  |
| Season |  |  | **0.13** | 0.10 |  |  | **0.03** | **0.01** |  |  | **0.01** | **0.03** |  |  | 0.26 | - |
| Spring | 7 (6.4%) | 103 (93.6%) |  |  | 88 (80.0%) | 22 (20.0%) |  |  | 87 (79.1%) | 23 (20.9%) |  |  | 2 (1.8%) | 108 (98.2%) |  |  |
| Summer | 0 (0.0%) | 25 (100.0%) |  |  | 16 (64.0%) | 9 (36.0%) |  |  | 14 (56.0%) | 11 (44.0%) |  |  | 2 (8.0%) | 23 (92.0%) |  |  |
| Fall | 0 (0.0%) | 94 (100.0%) |  |  | 58 (61.7%) | 36 (38.3%) |  |  | 77 (81.9%) | 17 (18.1%) |  |  | 1 (1.1%) | 93 (98.9%) |  |  |
| Winter | 2 (12.5%) | 14 (87.5%) |  |  | 10 (62.5%) | 6 (37.5%) |  |  | 9 (56.3%) | 7 (43.8%) |  |  | 0 (0.0%) | 16 (100.0%) |  |  |
| Education level | |  | **0.02** | **0.03** |  |  | 0.40 | - |  |  | **0.09** | **0.03** |  |  | 0.70 | - |
| ≤12 years | 2 (18.2%) | 9 (81.8%) |  |  | 9 (81.8%) | 2 (18.2%) |  |  | 6 (54.5%) | 5 (45.5%) |  |  | 0 (0.0%) | 11 (100.0%) |  |  |
| >12 years | 7 (3.0%) | 227 (97.0%) |  |  | 163 (69.7%) | 71 (30.3%) |  |  | 181 (77.4%) | 53 (22.6%) |  |  | 5 (2.1%) | 229 (97.9%) |  |  |
| Jobs |  |  | 0.92 | - |  |  | **0.12** | 0.24 |  |  | 0.86 | - |  |  | 0.69 | - |
| Any job | 6 (3.6%) | 161 (96.4%) |  |  | 112 (67.1%) | 55 (32.9%) |  |  | 128 (76.6%) | 39 (23.4%) |  |  | 3 (1.8%) | 164 (98.2%) |  |  |
| Homemaker | 3 (3.8%) | 75 (96.2%) |  |  | 60 (76.9%) | 18 (23.1%) |  |  | 59 (75.6%) | 19 (24.4%) |  |  | 2 (2.6%) | 76 (97.4%) |  |  |
| Type of current pregnancy | | | 0.70 | - |  |  | 0.49 | - |  |  | 0.69 | - |  |  | 0.47 | - |
| Spontaneous | 9 (3.8%) | 230 (96.2%) |  |  | 167 (69.9%) | 72 (30.1%) |  |  | 182 (76.2%) | 57 (23.9%) |  |  | 5 (2.1%) | 234 (97.9%) |  |  |
| Artificial | 0 (0.0%) | 6 (100.0%) |  |  | 5 (83.3%) | 1 (16.7%) |  |  | 5 (83.3%) | 1 (16.7%) |  |  | 0 (0.0%) | 6 (100.0%) |  |  |
| Parity |  |  | 0.29 | - |  |  | **0.00 ^c^** | **0.02** |  |  | **0.06** | 0.23 |  |  | **0.19** | 0.09 |
| 0 (nullipara) | 4 (2.6%) | 147 (97.4%) |  |  | 96 (63.6%) | 55 (36.4%) |  |  | 109 (72.2%) | 42 (27.8%) |  |  | 5 (3.3%) | 146 (96.7%) |  |  |
| ≥1 | 5 (5.3%) | 89 (94.7%) |  |  | 76 (80.9%) | 18 (19.2%) |  |  | 78 (83.0%) | 16 (17.0%) |  |  | 0 (0.0%) | 94 (100.0%) |  |  |

Data were presented as numbers and percentages. High or low trace element status groups were defined as a serum concentration outside the reference range. Reference ranges are: for serum cobalt concentration 0.13−0.73 μg/L, for serum copper concentration 55–150 μg/dL, for serum zinc concentration 65–125 μg/dL, and for serum concentration selenium 75–200 μg/L. No pregnant women with low cobalt or low copper or high zinc or high selenium were observed in our study population. Univariable analysis revealed that age was significantly associated with high copper and low zinc status (*p* < 0.01) and pre-pregnancy body mass index was associated with high copper status (*p* = 0.03). Multivariable analysis revealed that age was significantly associated with copper status (*p* = 0.04), zinc status (*p* < 0.01), and selenium status (*p* = 0.03) and pre-pregnancy body mass index was not associated with any of four trace elements status (*p* > 0.05). ^a^ *p* values for univariable analysis; ^b^ *p* values for multivariable analysis; ^c^ *p* < 0.01.


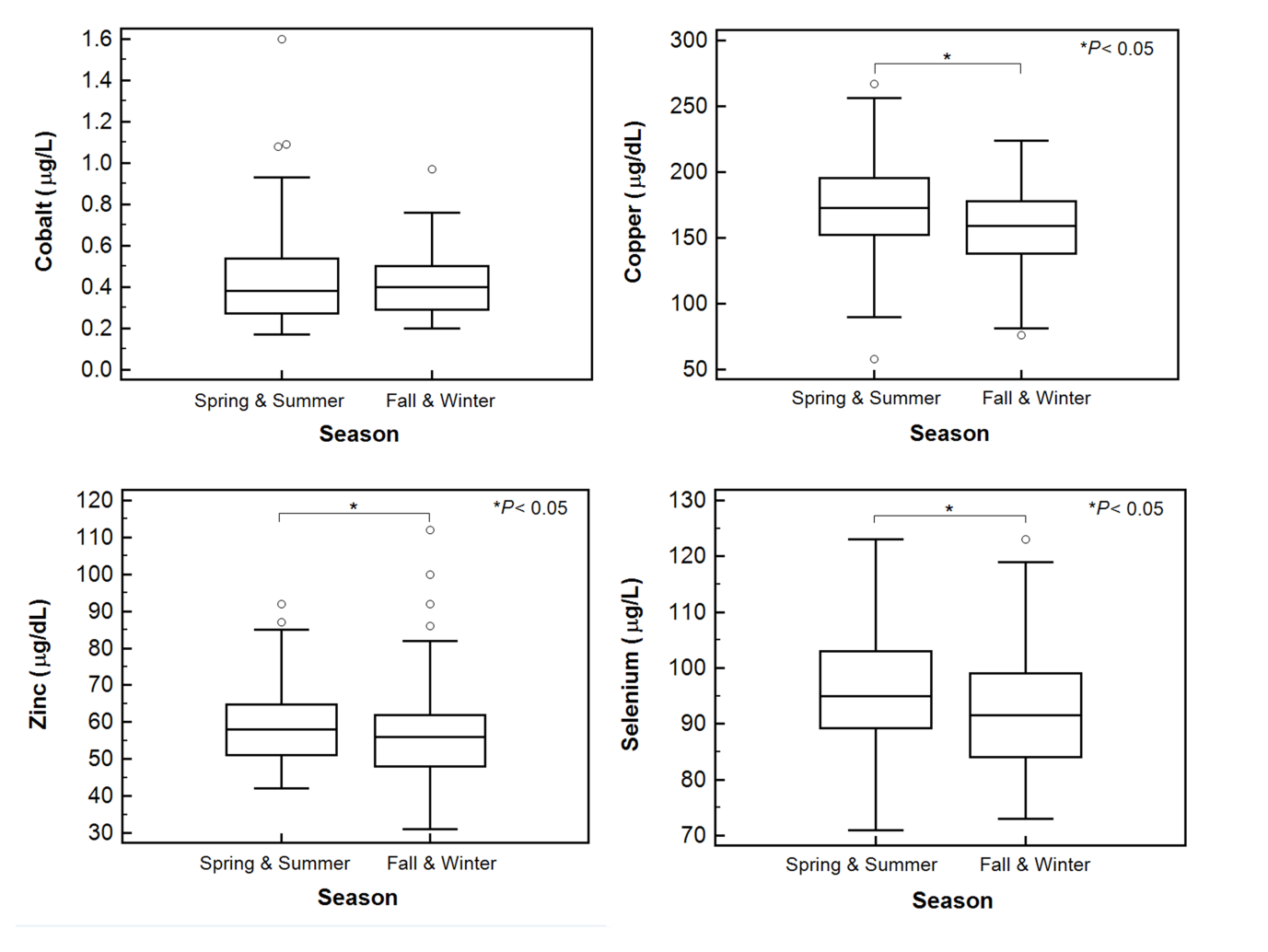


**Figure S1.** Trace element concentrations over the different seasons. Low copper, zinc, and selenium levels were also observed in fall and winter compared to in spring and summer.
